# The Role of Neural Signaling in the Pancreatic Cancer Microenvironment

**DOI:** 10.3390/cancers14174269

**Published:** 2022-08-31

**Authors:** Ryota Takahashi, Hideaki Ijichi, Mitsuhiro Fujishiro

**Affiliations:** Department of Gastroenterology, Graduate School of Medicine, The University of Tokyo, Tokyo 113-8655, Japan

**Keywords:** pancreatic ductal adenocarcinoma, tumor microenvironment, stroma, nerve

## Abstract

**Simple Summary:**

Pancreatic cancer is a highly lethal malignant disease with a dense stroma, called the tumor microenvironment. Accumulating evidence indicates the important role of sympathetic, parasympathetic, and sensory nerves in the tumor microenvironment of various cancers, including pancreatic cancer. Cancer cells and neural cells interact with each other to form a complex network and cooperatively promote cancer growth and invasion. In this review article, we describe the current understanding of the role of nerves in the tumor microenvironment.

**Abstract:**

Pancreatic cancer is one of the most lethal malignant diseases. Various cells in the tumor microenvironment interact with tumor cells and orchestrate to support tumor progression. Several kinds of nerves are found in the tumor microenvironment, and each plays an essential role in tumor biology. Recent studies have shown that sympathetic, parasympathetic, and sensory neurons are found in the pancreatic cancer microenvironment. Neural signaling not only targets neural cells, but tumor cells and immune cells via neural receptors expressed on these cells, through which tumor growth, inflammation, and anti-tumor immunity are affected. Thus, these broad-range effects of neural signaling in the pancreatic cancer microenvironment may represent novel therapeutic targets. The modulation of neural signaling may be a therapeutic strategy targeting the whole tumor microenvironment. In this review, we describe the current understanding of the role of nerves in the tumor microenvironment of various cancers, with an emphasis on pancreatic cancer. We also discuss the underlying mechanisms and the possibility of therapeutic applications.

## 1. Introduction

Pancreatic ductal adenocarcinoma (PDAC) is one of the deadliest cancers and is estimated to be the second leading cause of cancer-related deaths in the United States by 2040 [1]. Despite the advances in diagnosis and treatment, the 5-year survival rate still stands at 11% [2].

The tumor has a heterogeneous population of tumor cells and stromal cells called tumor microenvironment, which includes immune cells, fibroblasts, endothelial cells, extracellular matrix, and secreted factors [3]. The tumor microenvironment of PDAC is characterized by its dense stroma with various cells such as fibroblasts, immune cells, blood vessels, and nerves [4,5]. These cells in the tumor microenvironment and tumor cells interact with each other to form a complex network and support tumor progression by providing nutrition [6], growth factors, and cytokines/chemokines [7], suppressing anti-tumor immunity [8], and inhibiting efficient drug delivery [9].

The role of nerves in cancer has been implicated, because the infiltration of nerves in tumor stroma and neural invasion is often found in many cancers, including PDAC [10,11]. Recent studies have demonstrated the important roles of autonomic nerves such as sympathetic and parasympathetic nerves in the tumorigenesis of prostate cancer [12,13], ovarian cancer [14], gastric cancer [15,16], and basal cell carcinoma [17]. Accumulating evidence suggests interactions of nerves and various cells in the tumor microenvironment, including non-tumor cells. In this review, we provide an overview of the role of nerves in the tumor microenvironment, with an emphasis on PDAC.

## 2. Nerves in the Normal Pancreas and PDAC

Sympathetic and parasympathetic nerves innervate the pancreas [18]. Both exocrine and endocrine cells are regulated by sympathetic and parasympathetic nerve systems. Sympathetic nerve stimulation leads to a decrease in insulin and an increase in glucagon to maintain glycemic levels during stressful conditions [19,20,21]. Parasympathetic nerve activation increases insulin secretion [22]. The vagal nerve regulates pancreatic exocrine secretion [23]. In addition, sensory nerves also innervate the pancreas and may be involved in perceiving pain associated with chronic pancreatitis [24]. Neurotrophins, including nerve growth factor (NGF), brain-derived neurotrophic factor (BDNF), NT-3, and NT-4, play key roles in inducing nerve growth and axonal guidance in normal conditions [25]. For example, NGF is known to attract sympathetic and sensory nerves [26,27]. These molecules bind to different receptors, including the tropomyosin-related kinase (TRK) family of tyrosine receptor kinases and the low-affinity p75NTR [28].

**Figure 1:**
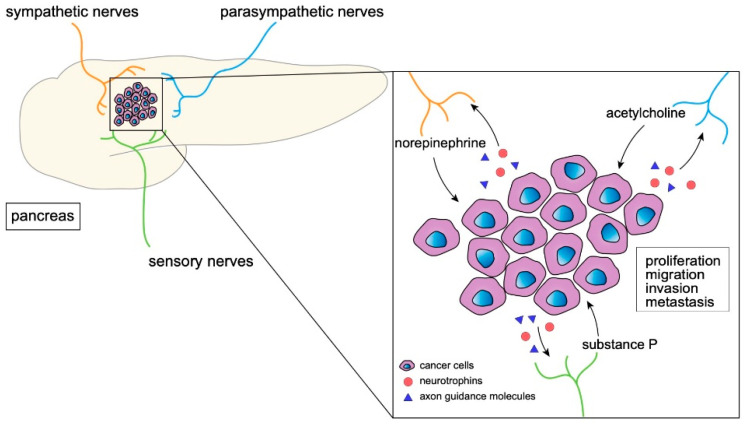
The interaction of neural cells and tumor cells. A schematic figure depicting the interaction of cancer cells and neural cells via various molecules.

Tumoral innervation is reportedly associated with patient prognoses in many cancers, such as breast [29,30,31,32], gastric [15,16], head and neck [33,34], ovarian [35], prostate [12,13,36,37,38], and pancreatic cancer [39,40,41,42,43]. Especially in the PDAC microenvironment, tumoral innervation is an important hallmark: increased neural density and hypertrophy compared with a normal pancreas was observed in a PDAC specimen and was associated with a poor prognosis [27,44]. These studies suggest a tumor-promoting interaction between nerves and cancer cells (Figure 1). In addition, perineural invasion is another important feature of PDAC, which is a disseminating process through lymphatic vessels along nerves supported by various cells in the perineural niche [45,46]. Intra- and extra-pancreatic perineural invasion by cancer cells is present in 70–100% of PDAC resection specimens and is associated with worse prognoses such as tumor recurrence and shorter patient survival [47,48]. Notably, the prevalence and severity of perineural invasion in PDAC were reported to be the highest among gastrointestinal malignancies [47], suggesting the highly neurotropic feature of PDAC.

## 3. The Effect of Neural Signaling on Tumor Progression

**Figure 2:**
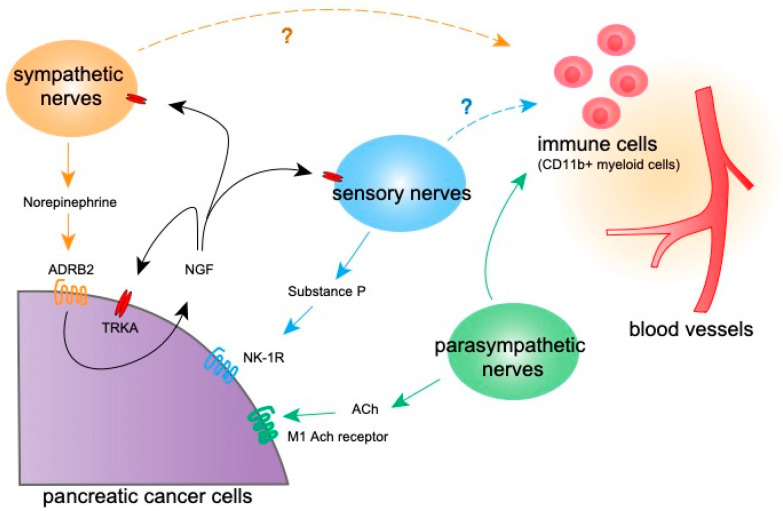
The signaling from nerves into pancreatic tumor microenvironment. A schematic figure showing signaling molecules from various nerves into pancreatic cancer cells and other components in the tumor microenvironment to regulate tumor progression. ADRB2, beta 2 adrenergic receptor. Ach, Acetylcholine. Question marks indicate effects reported in cancers other than PDAC.

Molecules released by various neural cells infiltrating the tumor microenvironment, such as neurotransmitters, have been shown to affect various aspects of tumor cell activity, such as migration, invasion, and metastasis [49,50]. Accumulating evidence suggests that nerves can directly promote cancer cell proliferation, as suggested by studies in which the co-culturing of dorsal root ganglia (DRG) and cancer cells led to the increased proliferation of prostate and PDAC cells [38,43]. Subsequent studies have shown that various molecules secreted from nerves affect both tumor and non-tumor cells in the tumor microenvironment (Table 1). PDAC is innerved by sympathetic nerves, parasympathetic nerves, and sensory nerves, which have different roles in the tumor microenvironment (Figure 2).

### 3.1. Sympathetic Nerves and Stress

The effect of sympathetic nerves on tumor development has been reported in various cancers. For example, chemical or surgical sympathectomy and the genetic deletion of stromal β2 and β3 receptors decreased prostate tumor development in mice [12], suggesting the role of sympathetic nerve signaling in early prostate tumorigenesis. Furthermore, it has been shown that β2 adrenergic signaling on tumor endothelial cells induces metabolic switch and promotes tumor angiogenesis [13]. Breast cancer growth and progression were accelerated following the stimulation of sympathetic nerves in tumors [29]. PDAC is also innervated by sympathetic nerves, and the surgical removal or pharmacological inhibition of β2 adrenergic signaling decreases PDAC development through the direct stimulation of tumor cells and indirect effects of the upregulated secretion of NGFs [39].

Catecholamines (norepinephrine and epinephrine) are neurotransmitters for sympathetic nerves. Sympathetic nerve signaling is mediated by α- and β-adrenergic receptors, which are widely expressed in the body. It has been shown that catecholamines stimulate ovarian cancer cells via β3-adrenergic receptors, and cancer cells, in turn, produce BDNF to promote tumor innervation, forming a feed-forward loop [35]. Gastric cancer growth and metastasis in mice were inhibited by the blockade of β2-adrenergic receptor signaling [51]. Similarly, catecholamines could induce PDAC cell proliferation, invasion, and perineural invasion through β-adrenergic signaling in vitro [52,53] and in vivo [54]. Specifically, PDAC cells are stimulated via β2-adrenergic receptors to exhibit increased invasion and proliferation [39,53], and PDAC cells, in turn, produce NGFs and BDNFs to promote tumor innervation [39]. The blockade of β2-adrenergic receptors suppressed tumor growth, tumor innervation, and perineural invasion, and prolonged the survival of mice with PDAC [39].

Catecholamines are induced by physiological and psychological stress, and epidemiological studies have suggested that stress is related to cancer incidence and tumor growth [84]. It has been reported that stress increases cancer mortality [85], and PDAC patients suffer from higher levels of stress than other types of cancers [86]. Stress has been shown to promote cancer progression in several cancer models, including ovarian cancer [14], prostate cancer [87], and PDAC [54,88], supporting the role of stress in tumor development and progression through adrenergic signaling. It has also been shown that stress induced by the housing temperature of mice bearing PDAC xenografts affected their sensitivity to cytotoxic therapies [89]. Interestingly, increased levels of stress in cancer patients were associated with cancer-related pain [90], suggesting the involvement of pain in increased levels of adrenergic signaling.

Thus, inhibitors or antagonists of adrenergic receptors might have inhibitory effects on tumor progression in a clinical setting. α- and β-adrenergic receptors are widely expressed on both normal and neoplastic cells, including in PDAC [91], and it has been reported that β-blockers may prolong the survival of patients suffering from colon cancer [84], breast cancer [92,93], ovarian cancer [94], melanoma [95], prostate cancer [96], and PDAC [97,98]. However, the effect of β-blockers on cancer prognosis seems to be tissue- or subtype-specific [99]. Thus, in future research, it is necessary to find the population in which β-blockers are the most effective.

Gamma-aminobutyric acid (GABA) is a molecule that negatively regulates β-adrenergic signaling. Although GABA has been reported to suppress PDAC cell proliferation [60], another study reported that GABA stimulated PDAC growth through overexpressing the GABA receptor pi subunit [61].

### 3.2. Parasympathetic Nerves

The role of parasympathetic nerves seems to be different depending on the type of cancer. In prostate cancer, it was reported that the stimulation of parasympathetic nerves increased tumor metastasis and invasion [12]. In this study, type 1 muscarinic receptor signaling in the stroma was shown to be critical for tumor progression, suggesting the importance of neural signaling in non-tumor cells in the tumor microenvironment. For gastric cancer, it was shown that vagal nerve signaling promoted gastric cancer through type 3 muscarinic-receptor-mediated Wnt signaling [16]. Similarly, type 3 muscarinic signaling promoted small cell lung carcinoma growth via mitogen-activated protein kinase (MAPK) and Akt signaling [71]. Type 3 muscarinic receptor signaling promoted the migration and invasion of colon cancer cells via the activation of matrix metalloproteinase-7 (MMP-7) and epidermal growth factor receptor [72].

In contrast, the stimulation of parasympathetic nerves decreased the tumor growth of breast cancer [29]. For PDAC, it has been suggested that vagal nerve activity, indexed by heart rate variability, is associated with the prolonged survival of metastatic PDAC patients [100]. Surgical vagotomy or parasympathetic nerve stimulation via type 1 muscarinic receptors resulted in suppressed pancreatic tumor development in mice [40,73], by inhibiting the release of tumor necrosis factor-α (TNFα) from macrophages, decreasing MAPK and phosphatidylinositol-3 kinase (PI3K) activity in tumor cells, and suppressing the expansion of cancer stem cell populations.

### 3.3. Sensory Neurons

Sensory neurons in the pancreas convey signaling related to pain. Contributions of sensory nerves to tumor progression have been reported in several studies. Sensory neuron ablation in a mouse model of PDAC induced by neonatal capsaicin injection prolonged the survival of the mice while suppressing inflammatory signals from the tumor to the central nervous system [41], suggesting the effect of sensory signaling from PDAC on immune systems. Head and neck squamous cell carcinomas are innerved by sensory neurons, which are promoted by exosomes containing an axon guidance molecule, EphrinB1, released from tumor cells [33]. Sensory denervation by surgery in a mouse model of oral cavity squamous cancer led to decreased tumor growth [34]. Several other studies have reported contributions of sensory nerves to tumor growth in cervical, skin, and thyroid cancers [17,101,102]. Substance P, a pain-associated tachykinin, and its high-affinity receptor NK-1R, are highly expressed in various cancer cells such as HER2-positive breast cancer, and contribute to cancer progression [75]. A subpopulation of pancreatic preneoplastic lesions expresses NK-1R, and substance P secreted by sensory neurons promoted tumor growth via the activation of JAK2 and STAT3 [76]. NK-1R is reported to also be expressed on tumor-associated blood vessels in various neoplasms [80]. Treating mice bearing a PDAC xenograft by substance P analogs decreased the tumor volume and angiogenesis [77]. Substance P is produced in both DRG neurons and PDAC cells, promoting the outgrowth of neurites and cancer cell proliferation and invasion [78,79]. Calcitonin gene-related peptide (CGRP) is another neuropeptide released from sensory neurons and has been shown to promote angiogenesis in lung cancer [81].

It has also been reported that sensory and sympathetic nerves in the PDAC microenvironment provide nutritional support to cancer cells by secreting serine and several other amino acids in serine/glycine-deprived conditions [83], suggesting a novel role of nerves in the tumor microenvironment. In cancer cells, glucose is processed via glycolysis and converted to pyruvate, then to lactate [103,104]. Some human PDACs lack an enzyme that converts glucose to serine and is thus are dependent on an external supply of serine to synthesize glycine, which enables the production of NGFs to increase tumor innervation [83].

## 4. The Effect of Neural Signaling on Non-Tumor Cells

As mentioned above, autonomic neural signals can not only affect tumor cells but also other types of cells, especially immune cells. The inflammatory status in the body is regulated via humoral and neuronal pathways [105,106,107]. For example, inflammatory responses to endotoxins are inhibited by vagal nerve stimulation and the release of acetylcholine [108].

### 4.1. Immune Cells

Associations between neuronal and immune systems have been reported to influence tumor immunity [109,110]. Neurogenic signatures were shown to be associated with immunosuppressive phenotypes [111].

The function of T cells, especially cytotoxic CD8^+^ T cells, is critical for anti-tumor immunity [112]. Some studies have suggested that neural signaling plays a role in controlling anti-tumor T cell functions. The ablation of sympathetic nerves decreased programmed death-1 (PD-1) and FOXP3 expression on T cells in breast cancer [29]. Accordingly, the parasympathetic stimulation of breast cancer cells decreased PD-L1 expression on tumor cells and PD-1 on T cells and increased CD8^+^/regulatory T (Treg) cells [29]. Another study demonstrated that the inhibition of β2-adrenergic receptor signaling on immune cells led to increased CD8^+^ T cells and decreased PD-1 expression on T cells [55]. In prostate cancer, PD-L1 expression on nerves in the tumor microenvironment was inversely correlated with the prevalence of CD8^+^ T cells and patient prognosis [113].

Macrophages infiltrating the tumor microenvironment are called tumor-associated macrophages (TAMs), which exert various effects to promote tumor initiation and progression [114]. In breast cancer, β-adrenergic nerve stimulation induces infiltration and the differentiation of tumor-promoting macrophages in the tumor microenvironment, leading to tumor progression and angiogenesis [56]. In contrast, cholinergic signaling suppresses the CD11b^+^ myeloid cell population and TNFα expression in the PDAC microenvironment, indicating the tumor suppressive and anti-inflammatory effect of cholinergic signaling [40]. Macrophages in the PDAC microenvironment are recruited by C-C chemokine receptor type 2 (CCR2) and colony-stimulating factors and secrete GDNFs to promote cancer migration and nerve invasion [115].

Myeloid-derived suppressor cells (MDSCs) are activated neutrophils and monocytes which have immune suppressive functions to promote tumor progression [116]. In melanoma, inhibition of β3-adrenergic receptor signaling attenuated regulatory T cells and MDSC increased the number and cytotoxicity of natural killer (NK) cells and increased the ratio of M1/M2 macrophages and N1 granulocytes [57]. Sensory neurons have been reported to secrete several CCL and CXCL chemokines in the melanoma microenvironment, attracting MDSCs to promote immune-tolerant conditions [82]. In colon cancer, cholinergic stimulation prevents colon cancer progression by inducing anti-inflammatory peptide trefoil factor 2 secretion from memory T cells to suppress MDSC expansion [74].

NK cells also play an important role in innate tumor immunity [117]. NK cells and nerves interact in the context of the degeneration of damaged sensory neurons through the NK cell receptor NKG2D and retinoic acid early-inducible 1 (RAE1) gene [118]. Due to β2 adrenergic receptor signaling, NK cells and other leukocytes are mobilized into circulation [119].

Eosinophils are granulocytes involved in innate immunity and have been shown to interact with neurons [120]. Nerves recruit eosinophils through the stimulation of neuropeptides, cytokines, and chemokines; eosinophils release cationic proteins, neurotrophins/neuropeptides, and ROS to induce nerve growth and neuropeptide synthesis. In the tumor microenvironment, the role of eosinophils seems to be context-dependent [121]. In some cancers, including melanoma, eosinophils exhibit anti-tumorigenic roles in mouse models, suggesting a novel therapeutic strategy.

### 4.2. Tumor Endothelial Cells (TECs)

Angiogenesis during tumor development has also been reported to be promoted by neural inputs. Vascular organization during development has been shown to be affected by sensory neurons [122], as well as signaling via neuropeptide Y [123]. In the tumor microenvironment, sympathetic nerve signaling induces angiogenesis, and TECs, in turn, promote tumorigenesis by secreting cytokines and growth factors [58]. Systemic sympathetic nerve stimulation by the chronic restrain model revealed increased vascular endothelial growth factor (VEGF) expression and angiogenesis via β2-adrenergic receptor signaling in ovarian tumor cells [14]. Catecholamines signaling through β-adrenergic receptors also induced expression of VEGF and IL-6 in breast cancer cells [59]. In prostate cancer, β2-adrenergic receptor signaling on endothelial cells promoted tumor angiogenesis and tumor progression [13], suggesting a mechanism involving immune regulation by sympathetic nerves through endothelial cells. In addition, catecholamine treatment induced the alternatively activated M2 polarization of macrophages to secrete VEGF and promote tumor angiogenesis in a lung cancer mouse model [124]. On the other hand, dopamine, a neurotransmitter of sympathetic nerves, downregulates VEGF receptor 2 signaling in endothelial cells [62] and inhibits colon cancer angiogenesis and growth [63]. Similarly, gastric cancer and ovarian cancer mouse models have shown decreased tumor angiogenesis and tumor growth after dopamine treatment [64,65]. Collectively, in the tumor microenvironment, sympathetic innervation promotes angiogenesis supporting tumor progression.

### 4.3. Cancer-Associated Fibroblasts (CAFs)

CAFs are key components in the tumor microenvironment and have been extensively investigated and shown to have various functions, including modifying matrix deposition, reciprocal signaling, and interacting with cancer cells and immune cells to promote cancer progression [125]. CAFs have also been shown to secrete several axon-guidance molecules. Exosomes derived from head and neck cancer cells induced NGF expression in fibroblasts [126]. Pancreatic stellate cells also produce neurotrophic factors NGFs and artemin in response to transforming growth factor β (TGF-β) to induce neurite outgrowth [127,128,129]. In pancreatic cancer, CAFs expressing NetrinG1, an axon-guidance molecule, have been shown to metabolically support tumor growth by affecting glutamate/glutamine metabolism and inhibiting NK-cell-mediated tumor killing via the Akt and p38 pathways [130]. CAFs in the pancreatic cancer microenvironment have been reported to secrete an axon guidance molecule, SLIT2, to induce neural outgrowth [131]. These studies suggest that CAFs are an important mediator of tumor innervation and neural remodeling in the tumor microenvironment.

### 4.4. Cancer-Associated Adipocytes(CAAs)

In adipocytes, β-adrenergic signaling, especially β3, is involved in the lipolytic mobilization of fatty acids [132,133]. In the cancer microenvironment, CAAs have been reported to promote tumor growth, angiogenesis, and migration through the secretion of hormones, cytokines, adipokines, and growth factors [134].

## 5. Origins of Nerves in the Tumor Microenvironment

The mechanism of how neural cells expand in the tumor microenvironment is not clearly understood. One possibility is that pre-existing nerves directly innervate from the surrounding tissue. Co-culturing neural ganglia and cancer cells promote neurite outgrowth [38,39]. Such innervation might be induced by neurotrophins including nerve growth factors. Another possibility is the trans-differentiation of cells in the tumor microenvironment. Amit and colleagues reported that loss of *TP53* in oral cancer induced the trans-differentiation of tumor-associated sensory neurons into adrenergic neurons [34]. Another study suggested the possibility of the trans-differentiation of cancer cells into neural cells in the tumor microenvironment of prostate cancer [135]. Lastly, neural progenitor cells might be recruited to the tumor microenvironment from distant organs. Mauffrey and colleagues reported that doublecortin (DCX)-positive neural progenitors from the central nervous system infiltrated prostate primary tumors and metastases [136].

## 6. The Molecular Mechanisms Involved in Nerve Expansion in the Tumor Microenvironment

**Figure 3:**
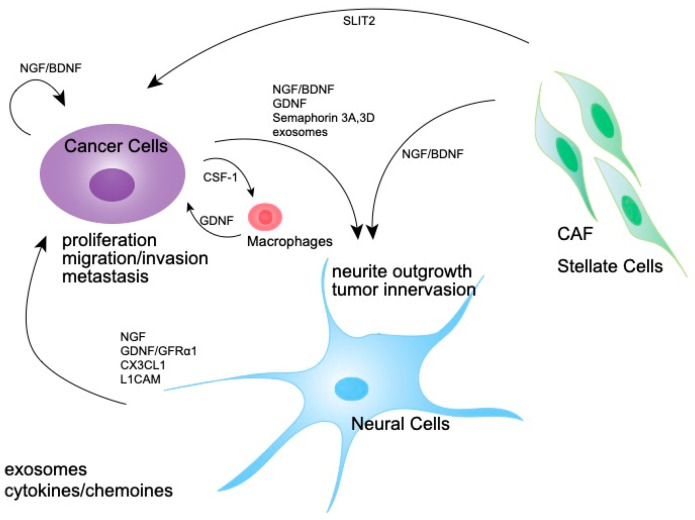
Molecular mechanism of the expansion of tumor-associated nerves. A schematic diagram showing the interaction of nerves, pancreatic cancer cells, and CAFs via neurotrophins and other molecules which induce nerve growth.

Various molecules, including neurotrophins, axon guidance molecules, and cytokines, are reportedly involved in the development and function of nerves in the tumor microenvironment (Figure 3).

### 6.1. Neurotrophins

Nerve growth is physiologically mediated by molecules such as neurotrophins [25], axon guidance molecules [137], and other growth factors. In cancer development, in addition to neural cells, cancer cells aberrantly produce neurotrophins to promote further innervation into the tumor microenvironment in various cancers, including breast cancer and PDAC [10,15,30,35,39,138,139,140]. Recent studies have shown that neurotrophins also induce the proliferation, migration, and invasion of tumor cells, including breast, colon, ovarian, and prostate cancer, and PDAC [10,138,141,142,143,144,145,146,147,148,149]. Thus, neurotrophins released from tumor cells can induce both tumor innervation in a paracrine manner and tumor progression in an autocrine manner, whereas Schwann cells and nerves also release NGFs and glial cell line-derived neurotrophic factors (GDNFs) to facilitate cancer progression in nerves [66,67]. In addition, pancreatic stellate cells were reported to secrete NGFs [127] and BDNFs [128], increasing neural density in DRG in vitro [129].

The expression of NGF or its precursor, proNGF, and its receptors have been associated with reduced survival in several cancers [36,141,150,151]. NGF depletion by anti-NGF antibodies [152], NGF siRNA [153], or an antagonist of TRK receptors [39] reduced progression, metastasis, tumor innervation, and prolonged survival in mouse models of PDAC. TRKB, a high-affinity receptor for BDNF shows increased expression on metastatic PDAC cells [154] and is associated with higher invasion [145]. NT-3 is reported to be expressed mainly in the stroma of PDAC [145], whereas its receptor, TRKC, was expressed on PDAC cells and intratumoral nerves [155]. Blocking NT-3 suppressed the growth of PDAC in a xenograft mouse model [146].

### 6.2. GDNFs

GDNF family members [156] are axon guidance molecules, consisting of GDNF, nerturin (NRTN), artemin (ARTN), and persephin (PSPN). These molecules are reported to be associated with advanced diseases and perineural invasion in PDAC [67,68,157,158,159]. Studies suggest that GDNF family receptor α1 (GFRα1) is released from nerves and facilitates the binding of nerves and PDAC cells via GDNF–RET interactions [68,69]. Interestingly, tumor-associated macrophages also secrete GDNFs, which stimulate RET on cancer cells to promote perineural invasion [115].

### 6.3. Semaphorins

Semaphorins are a family of axon guidance molecules [160]; some semaphorins have been associated with tumor innervation. Semaphorin 4F overexpression in prostate cancer cells induces cancer-related neurogenesis [38]. In PDAC, frequent copy number variations and mutations of semaphorin 3A and 3E have been observed [161]. Semaphorin 3A was found to be expressed in PDAC cells and nerves in cancerous specimens and is associated with poor prognoses [162]. Semaphorin 3D, secreted by PDAC cells, acts on Plexin D1 on neural cells to induce tumor innervation and proliferation [42]. Other semaphorin family members, such as semaphorin 3E [163], 5A [164], and 6C [165] were also reported to affect PDAC progression, although the involvement of nerves in the tumor microenvironment was not demonstrated.

### 6.4. SLIT/ROBO Signaling

Cancer-associated fibroblasts (CAFs) are suggested to be another source of neurotrophins and axon guidance molecules [126,130]. CAFs in PDAC secrete an axon guidance molecule, SLIT2, which induces repulsion and enhanced migration of neural cells [166], and takes part in neural remodeling in the tumor microenvironment [131]. SLIT/ROBO signaling has been suggested to be required to preserve pancreatic cell identity [167]. In PDAC, frequent mutations or copy number losses of SLIT2, ROBO1, and ROBO2 were identified in human PDAC analyses [161]. In addition, lower levels of expression of ROBO2 were associated with poor prognoses in PDAC patients [161]. Another study confirmed that SLIT2 expression was reduced in PDAC, and showed that restoring SLIT2 inhibited the neural invasion and metastasis of PDAC [168], suggesting its suppressive role in nerve–cancer interactions.

### 6.5. Cell Adhesion Molecules

Neural cell adhesion molecule 1 (NCAM1) is expressed in neurons and developing Schwann cells, and helps neural growth, adhesion, and regeneration [169]. NCAM expression is correlated with neural invasion [170] and decreased survival in PDAC [171]. In addition, it has been suggested that NCAM1 expressed on Schwann cells could promote the migration and dispersion of cancer cells [66].

L1 cell adhesion molecule (L1CAM) is another adhesion molecule expressed on neural cells. L1CAM is highly expressed in PDAC cells, the expression levels of which are correlated with cancer progression, metastasis, and neural invasion via the induction of metalloproteinase-2 (MMP-2) and MMP-9 in cancer cells [172,173,174,175].

### 6.6. Cytokines/Chemokines and Exosomes

Some cytokine/chemokines have been shown to induce tumor innervation. Granulocyte-colony stimulating factor (G-CSF) was reported to suppress sympathetic nerve damage and promote parasympathetic nerve growth in the prostate cancer microenvironment [37]. Colony-stimulating factor (CSF-1) and CCL2–CCR2 axis were also reported to attract macrophages or monocytes and promote cancer cell migration and nerve invasion [115]. CCL is also released from nerves to induce the neural invasion of prostate cancer [176]. CX3CL1 is expressed in neural cells to act on PDAC cells through CX3CR1 and promote neural invasion and dissemination along nerves [70].

Exosomes, which are small, membrane-bound vesicles and contain molecules such as proteins, lipids, DNAs, and RNAs, are reportedly secreted from tumor cells to affect various cells in the tumor microenvironment [177]. Tumor–nerve interactions may be mediated by exosomes, as shown in one study where Madeo and colleagues reported that EphrinB1 is released from head and neck cancer cells packaged in exosomes and induces tumor innervation [33]. Exosomes were also reported to mediate innervation in cervical carcinoma [101,178]. More recently, miR-34a-3p contained in extracellular vesicles has been shown to suppress the trans-differentiation of tumor-associated sensory nerves into sympathetic nerves in head and neck cancer [34].

## 7. Clinical Applications of Nerve-Targeting Therapy

Revealing the molecular mechanisms underlying nerves in the tumor microenvironment leads to novel therapeutic targeting, although only a limited number of molecular-targeting drugs have been approved in the field of tumor-associated nerves. Inhibitors of TRK receptors (pan-TRK inhibitors; entrectinib and larotrectinib) have been approved for solid tumors with TRK fusion [179,180,181]. The effect of a multi-kinase inhibitor sitravatinib, which also inhibits Trk activity, on advanced solid tumors is currently being investigated (NCT02219711) [182]. Although these drugs target TRK receptor signaling in cancer cells, they may exert inhibitory effects on innervation and tumor–nerve interactions in the tumor microenvironment, which should be determined in future studies.

Clinical trials to examine the effects of muscarinic agonists on PDAC (NCT03572283) and β-blockers in both non-metastatic and metastatic prostate and pancreatic cancer patients (NCT02944201, NCT03152786, NCT03838029, and NCT04245644) are ongoing. In addition, NK-1R antagonists have been suggested to exert anti-cancer effects both in a pre-clinical and clinical setting [183]. Although these studies mainly target neural signaling in cancer cells, autonomic nerve signaling may also affect other targets including immune systems. In addition, CCR2 inhibitor treatment has been reported to enhance anti-tumor immunity in PDAC [184], and has shown tolerability in PDAC patients [185], possibly also having inhibitory effects on neural invasion or tumor innervation.

The most commonly observed serious treatment-related events were cognitive disorders in entrectinib [180], suggesting a possible adverse effect on neural cells outside tumors by nerve-targeting drugs; moreover, muscarinic agonists or β-blockers may affect the cardiovascular function and bowel movements via autonomic nerve signaling. As such, for novel therapeutic agents targeting nerves, attention should be focused on avoiding adverse effects on normal neural activity.

Future studies should test multiple pathways and interactions in the tumor microenvironment discussed above. Surgical or pharmacological denervations of sympathetic/parasympathetic nerves or sensory neurons, and targeting immune–nerve or CAF–nerve interactions, may warrant future clinical studies. Overcoming suppressed anti-tumor immunity by modulating neural signaling may pave the way for novel immunotherapies in PDAC or other immunologically “cold” tumors. Metabolites secreted from nerves may also be an important therapeutic target, as the PDAC tumor microenvironment places a high demand on nutrients and might be dependent on the continuous supply of metabolites from stroma, including nerves.

Due to the complexity of the heterogeneity in the tumor microenvironment, determining an appropriate target is a critical process for developing novel therapeutic agents. Considering the higher incidence of perineural invasion and its impact on patient prognosis [47,48], it is important to elucidate the PDAC-specific mechanism by which PDAC becomes a more neurotropic tumor. One possibility may be the contribution of desmoplastic stroma, which characterizes PDAC. CAFs in PDAC have been shown to be a highly heterogeneous population [186]; thus, subpopulations of CAF may provide niche factors to maintain and expand neural cells and facilitate interactions between tumors and neural cells.

## 8. Conclusions

Due to the complex interactions between nerves and multiple types of cells in the tumor microenvironment, developing treatments targeting nerves has proven difficult. Recent studies have revealed the important role of nerves in the tumor microenvironment; therefore, nerves and neural signaling seem to be attractive therapeutic targets in PDAC, which could have multi-dimensional effects such as tumor inhibition, immune modulation, and controlling angiogenesis. The close cooperation of researchers and physicians is vital to further understanding the mechanism underlying nerves in the tumor microenvironment and the development of novel and effective therapeutic options.

## Figures and Tables

**Table 1:** Molecules secreted by nerves and their effects on target cells.

Type of Nerves	Name of Molecules	Target Cells	Effect	References
sympathetic nerves	norepinephrine, epinephrine	cancer cells	tumor progression	[29,35,39,51,52,53,54]
		immune cells	immune suppression	[29,55,56,57]
		endothelial cells	angiogenesis	[13,14,58,59]
	GABA	cancer cells	tumor suppression	[60]
			tumor progression	[61]
	dopamine	endothelial cells	suppression of angiogenesis	[62,63,64,65]
	NGF, BDNF	cancer cells	tumor progression	[66,67]
	GFRα1	cancer cells	tumor progression	[68,69]
	CX3CL1	cancer cells	tumor progression	[70]
parasympathetic nerves	acetylcholine	cancer cells	tumor progression	[12,16,71,72]
		cancer cells	tumor suppression	[29,40,73]
		immune cells	immune activation	[29,40,74]
sensory nerves	substance P	cancer cells	tumor progression	[34,75,76,77,78,79]
		endothelial cells	suppression of angiogenesis	[77,80]
	CGRP	endothelial cells	angiogenesis	[81]
	CCL/CXCL chemokines	immune cells	immune suppression	[82]
sympathetic/sensory nerves	serine	cancer cells	tumor progression	[83]
